# A Bibliometric Analysis and Visualization of the Top-Cited Publications in Mild Traumatic Brain Injury

**DOI:** 10.3389/fneur.2021.687796

**Published:** 2021-06-09

**Authors:** Jian Shi, Xianping Du, María José Cavagnaro, Na Li

**Affiliations:** ^1^Department of Spine Surgery, The Third Xiangya Hospital, Central South University, Changsha, China; ^2^Department of Mechanical and Aerospace Engineering, Rutgers University, Piscataway, NJ, United States; ^3^College of Medicine, University of Arizona, Tucson, AZ, United States; ^4^Department of Radiology, Third Xiangya Hospital, Central South University, Changsha, China

**Keywords:** mild traumatic brain injury, top-cited publications, bibliometric analysis, visualization, neurotrauma

## Abstract

**Background:** For measuring their impact in scientific research, the citation count of the publications is used in the bibliometric analysis, though still in the bibliometric analysis, there is no comprehensive summary of mild traumatic brain injury (mTBI) research. This article intends to provide the physicians and the neuroscientists with a reference guide to assess the most influential publications written on this subject through a macroscopic view of the research activities on mTBI.

**Methods:** The database of the Web of Science was used to compile the 100 top-ranking publications on mTBI. The selected publications were evaluated on the basis of the several categorizations including the type of the publications, number of citations, country of origin, and year of publication.

**Results:** Between 1946 and 2020, the 13,040 publications that were published were included in the database. The least cited publications received 274 citations, while the most cited received 1,748. Altogether, 71 publications were from the USA while 29 were from other countries. Among all the institutions, the University of Pittsburgh Medical Center led the list with six publications. Around 100 papers, mostly on the clinical studies in the categories of neurology and neurosciences, were published in 54 different journals.

**Conclusions:** This study provides both a transverse section summary and historical retrospect for the clinical advances of mTBI, and the publications of important observations that contributed a significant impact on the treatment and prevention of mTBI had been identified.

## Introduction

In the United States with the incidence of around 2.5 million emergency department visits per year, Mild traumatic brain injury (mTBI) is known to be the most common form of traumatic brain injury (1). With the deepening of research on mTBI, more and more academic papers have been published. The information in the database will also be more complex, making it increasingly difficult for clinicians to find the most needed and more valuable research. Therefore, requirements for improving the deep understanding of mTBI, decreasing the morbidity, and improving the management of mTBI are indispensably needed.

Citation rate is an important index to quantify the quality of publications and research, although the number of citations for one article cannot completely reflect the level and academic quality of the research, indicating in its category, how celebrated that article has been (2, 3). Based on the number of quantitative laws, in the analysis of research literature in information sciences, bibliometrics has become a certified method. It is being extensively used in multiple disciplines and clinical specialties, including cardiology, gastroenterology, anesthesiology, urology, orthopedics, oncology, and obstetrics/gynecology (4–10). However, bibliometric analysis has not yet been used to the field of mTBI. Hence, this study has been intended to render insights and perspectives in the field of mTBI of the most cited publications. The evolving trends in highly cited articles could enable the researchers to access vital information regarding the requisite citation classics in the field and the overall development in this field around the world could also be understood and promoted to a certain extent by the bibliometric analysis of mTBI.

## Methods

**Figure 1:**
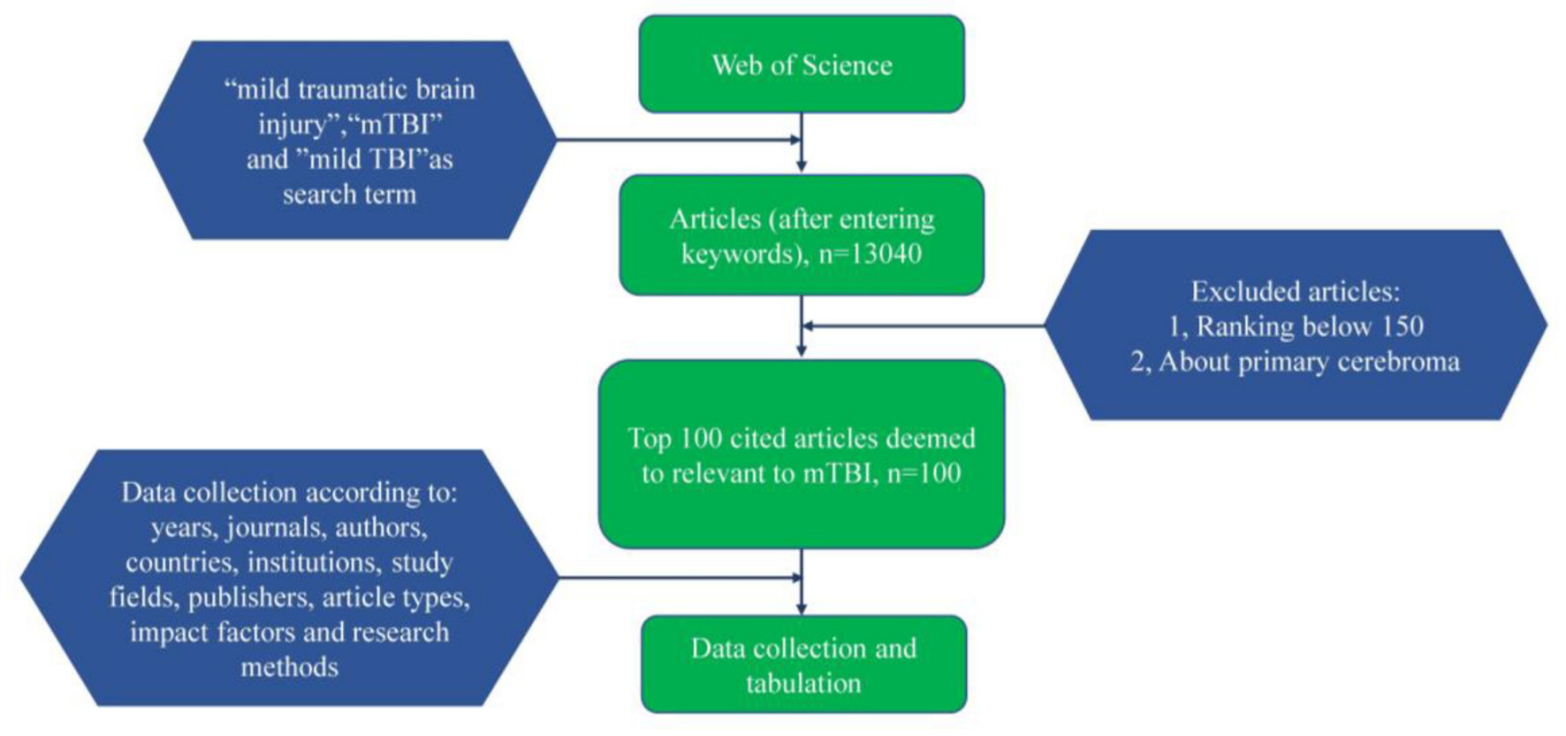
Flow chart showing the methodology used in the study.

Three independent researchers on June 4, 2020 made use of the Web of Science (WOS), the most professional and authoritative citation indexing database, to recognize the 100 top-cited publications in the field of mTBI. The keywords “mTBI,” “mild TBI,” or “mild traumatic brain injury” were used to search the database. Publications specific to mTBI were identified. There were no restrictions on the article types. The 100 top-cited publications in the field of mTBI were identified and reviewed. As illustrated in Figure 1, all keywords were continuously filtered and finally stopped at 13,040 where the number of publications stabilized, to ensure the relevance and the breadth of the search.

After confirming the search scope, next comes the recognition of the 100 top-cited publications from the 13,040 publications, which were arranged in reverse chronological order. Then, screening of the relevant publications was done by three independent researchers. After reading the full text and abstract of each selected article, data collection was completed according to: years, journals, authors, countries (refer to corresponding authors' affiliations), institutions, study fields, publishers, article types, impact factors, and research methods. By logging into certain other search platforms like PubMed, Sciencedirect, Springerlink, and BioOne, publications without complete information and full text were obtained. In addition, we excluded publications (a) ranking below 150 and (b) about primary cerebroma such as pituitary adenomas, schwannomas, pineocytomas, and etc. according to the selection criteria. Contributions to the design and concept of the study were made by all the authors.

## Results

### The Basic Characteristics of the 100 Top-Cited Publications

**Figure 2:**
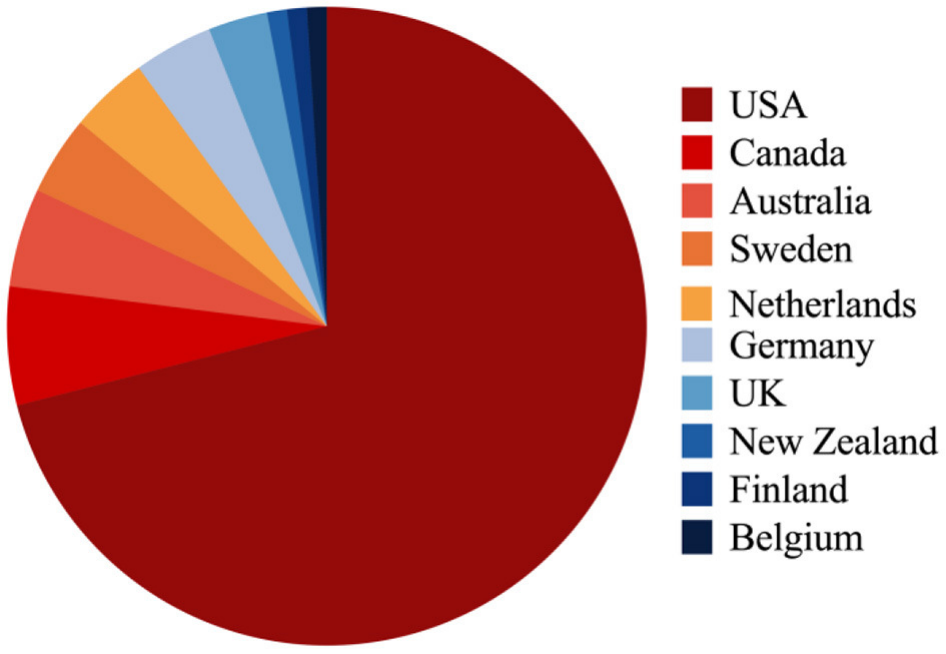
Country distribution of the top-cited articles.

**Figure 3:**
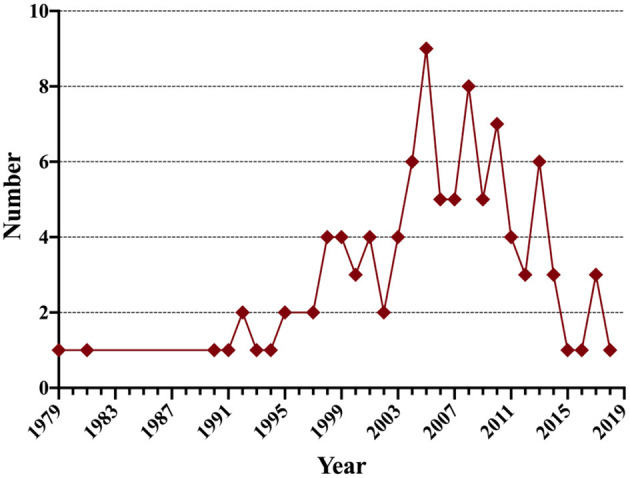
Distribution of the top-cited articles per year.

The total number of literatures extracted from the Web of Science was 13,040. After our screening, we reached results that the article with the largest citation number, 1,749 times, was an epidemiology research on a combat-related mild traumatic brain injury in about 2,525 U.S. Army infantry soldiers published in 2008 in the New England Journal of Medicine (“Mild traumatic brain injury in US Soldiers returning from Iraq”). In the Annals of Emergency Medicine, the article in 2008 on a clinical policy research on evidence-based recommendations of select issues in the management of adult mild traumatic brain injury was the one with the least citations numbering 262 (“Clinical policy: neuroimaging and decision-making in adult mild traumatic brain injury in the acute setting”). These publications were published between 1946 and 2020. Distribution of the top-cited publications per year is shown in Figure 1. With close to three quarters of the publications being contributed by the most prolific country, of the 100 publications from 10 different countries, the USA led by contributing 72 publications followed by Canada (*n* = 17), Australia (*n* = 5), Sweden (*n* = 4), Netherlands (*n* = 4), Germany (*n* = 4), UK (*n* = 3), and other countries (Table 1, Figure 2). And the distribution of the top-cited publications per year was shown in Figure 3.

**Table 1:** Country distribution of the top-cited articles.

**Country**	**Record count**
USA	71
Canada	6
Australia	5
Sweden	4
Netherlands	4
Germany	4
UK	3
New Zealand	1
Finland	1
Belgium	1
Total	100

### The Most Cited Publications in mTBI

The 100 top-cited publications were cited 277.6 times on average and the top 10 most-cited publications on mTBI are presented in Table 2 with the name of the first author, impact factor, published year, country, and total citations.

**Table 2:** The Top 10 most-cited articles on mTBI.

**Rank**	**Title**	**First author**	**Journal (IF 2019)**	**Year**	**Country**	**Citations**
1	Mild traumatic brain injury in US soldiers returning from Iraq	Hoge, CW.	New England Journal of Medicine (74.699)	2008	USA	1,749
2	Treatment of traumatic brain injury with moderate hypothermia	Marion, DW.	New England Journal of Medicine (74.699)	2007	USA	939
3	The spectrum of disease in chronic traumatic encephalopathy	McKee, AC.	Brain (11.337)	2013	USA	931
4	Long-Term cognitive impairment after critical illness	Pandharipande, PP.	New England Journal of Medicine (74.699)	2013	USA	922
5	Acute effects and recovery time following concussion in collegiate football players—The NCAA concussion study	McCrea, M.	JAMA-Journal of The American Medical Association (45.54)	2003	USA	904
6	Neuronal and glial apoptosis after traumatic spinal cord injury	Liu, XZ.	The Journal of neuroscience (6.074)	1997	USA	746
7	Incidence, risk factors and prevention of mild traumatic brain injury: Results of the WHO collaborating center task force on mild traumatic brain injury	Cassidy, JD.	Journal of Rehabilitation Medicine (2.046)	2004	Canada	737
8	Prognosis for mild traumatic brain injury: Results of the WHO collaborating center task force on mild traumatic brain injury	Carroll, LJ.	Journal of Rehabilitation Medicine (2.046)	2004	Canada	731
9	A systematic review of brain injury epidemiology in Europe	Tagliaferri, F.	Acta Neurochirurgica (1.817)	2006	USA	725
10	Evidence-based cognitive rehabilitation: Updated review of the literature from 1998 through 2002	Cicerone, KD.	Archives of Physical Medicine and Rehabilitation (3.098)	2005	USA	716

### Journals, Authors, and Institutions

**Figure 4:**
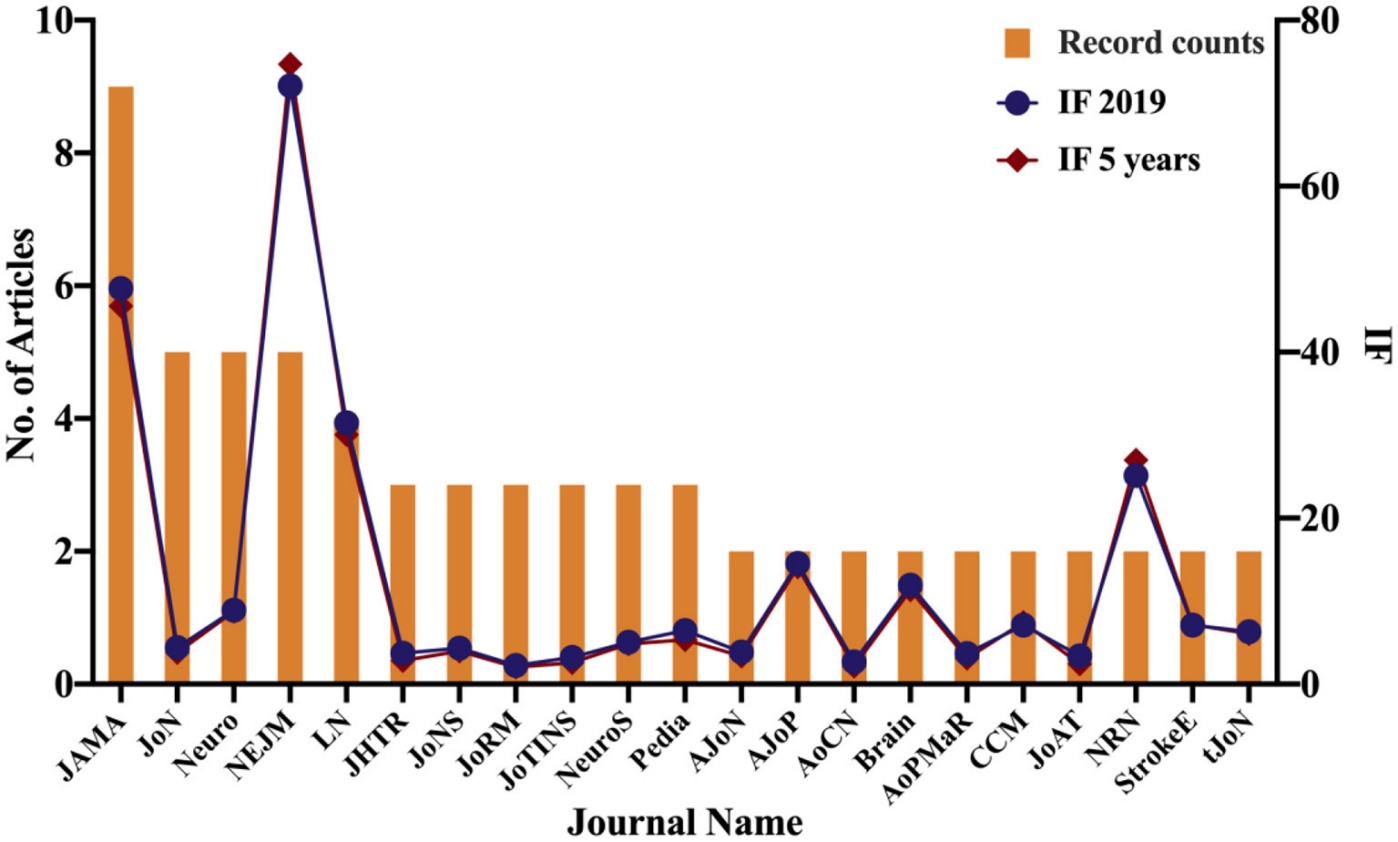
Journals with more than 2 top-100 cited articles.

Table 3, Figure 4 enlist the journals of the 100 top-cited publications in a descending order by the impact factor and the average number of citations per paper (2019/last 5 years). Altogether, 54 journals were included with the leading of: JAMA-Journal of The American Medical Association (*n* = 9), New England Journal of Medicine (*n* = 5), Neurology (*n* = 5), Journal of Neurotrauma (*n* = 5), Lancet Neurology (*n* = 4), and others (*n* = 72).

**Table 3:** Journals with more than 2 top-100 cited articles.

**Rank**	**Journal name**	**No. of articles**	**IF (2019)**	**IF (last 5 years)**
1	JAMA-Journal of The American Medical Association	9	45.54	47.677
2	New England Journal of Medicine	5	74.699	72.098
3	Neurology	5	8.77	8.899
4	Journal of Neurotrauma	5	3.793	4.417
5	Lancet Neurology	4	30.039	31.504
6	Journal of Head Trauma Rehabilitation	3	2.814	3.721
7	Journal of Neurosurgery	3	3.968	4.33
8	Journal of Rehabilitation Medicine	3	2.046	2.236
9	Journal of The International Neuropsychological Society	3	2.576	3.208
10	Neurosurgery	3	4.853	5.047
11	Pediatrics	3	5.359	6.45
12	American Journal of Neuroradiology	2	3.381	3.853
13	American Journal of Psychiatry	2	14.119	14.542
14	Archives of Clinical Neuropsychology	2	2.154	2.666
15	Brain	2	11.337	11.931
16	Archives of Physical Medicine and Rehabilitation	2	3.098	3.69
17	Critical Care Medicine	2	7.414	7.114
18	Journal of Athletic Training	2	2.416	3.394
19	Nature Reviews Neurology	2	27	25.125
20	Stroke	2	7.19	7.113
21	The Journal of Neuroscience	2	6.074	6.302

Listed as the first authors, there were around 10 authors who had contributed more than two top-cited publications. Polderman K H, the author of four of the top 100 publications led the list. Nine other authors, with two of the top 100 publications each, followed. Table 4 enlists these 10 authors.

**Table 4:** Authors with two or more top-cited articles.

**Author name**	**No. of articles**
Polderman, KH	4
Omalu, BI	2
Povlishock, JT	2
McAllister, TW	2
Marion, DW	2
Johnson, VE	2
Guskiewicz, KM	2
Carroll, LJ	2
Belanger, HG	2
Bazarian, JJ	2

The institution with the largest number of publications was led by the University of Pittsburgh Medical Center (*n* = 6), followed by the Virginia Commonwealth University (*n* = 4). The remaining different institutions like the University of California Los Angeles, Columbia University, Cornell University Weill Medical College, Dartmouth-Hitchcock Medical Center, James A. Haley Veterans' Hospital, The Royal Children's Hospital (Melbourne), and The University of Texas, Galveston, USA all produced two publications, respectively (Table 5).

**Table 5:** Institutions with more than two top-100 cited articles.

**Institution**	**Record count**
University of Pittsburgh Medical Center, Pittsburgh, USA	6
Virginia Commonwealth University, Richmond, USA	4
University of California, California, USA	2
University of California, Los Angeles, USA	2
Boston University, Boston, USA	2
Columbia University, New York, USA	2
Cornell University Weill Medical College, New York, USA	2
Dartmouth-Hitchcock Medical Center, Lebanon, USA	2
James A. Haley Veterans' Hospital, Florida, USA	2
The Royal Children's Hospital, Melbourne, Australia	2
The University of North Carolina, Chapel Hill, USA	2
The University of Texas, Galveston, USA	2
University Medical Center Utrecht, Utrecht, Netherlands	2
University of Alberta, Edmonton, Canada	2
University of Pennsylvania, Philadelphia, USA	2
University of Rochester, New York, USA	2
University of Washington, Seattle, USA	2
Vrije Universiteit Amsterdam, Amsterdam, Netherlands	2
Walter Reed Army Institute of Research, Maryland, USA	2
Washington University, St. Louis, USA	2

### Article Types, Research Methods, and Categories

**Figure 5:**
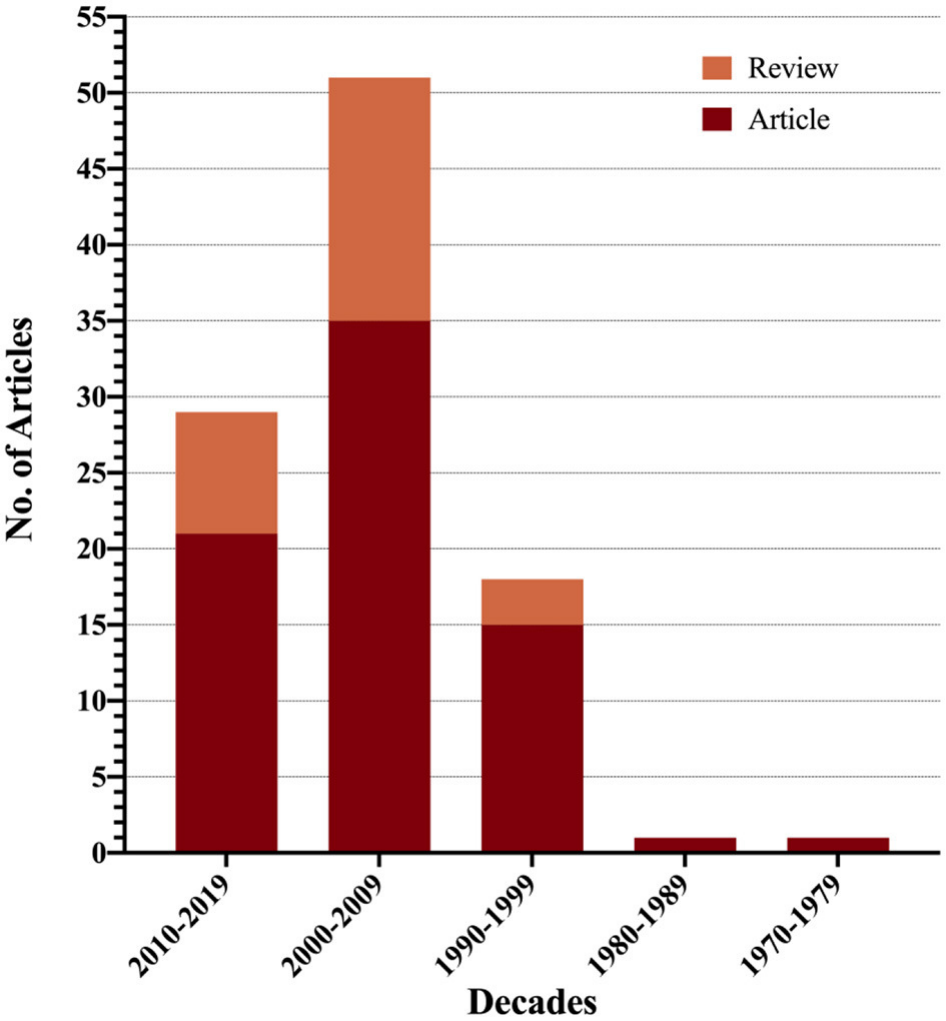
Article types vs. decades.

Figure 5 indicated the top 100 most-cited publications with decades while Table 6 illustrated the research methods in them. The research methods of the top 100 most-cited publications could be categorized into clinical study, review, basic science research, epidemiologic research, meta-analysis, questionnaires and classification, and other observational cohort study. Clinical study Etiology took the largest proportion of the top 100 most-cited publications (*n* = 49), followed by review (*n* = 25), basic science research (*n* = 10), and Epidemiologic research (*n* = 8).

**Table 6:** Research methods.

**Method**	**Record counts**
Clinical study	49
Review	25
Basic science research	10
Epidemiologic research	8
Meta-analysis	4
Questionnaires and classification	2
Other observational cohort study	2

**Figure 6:**
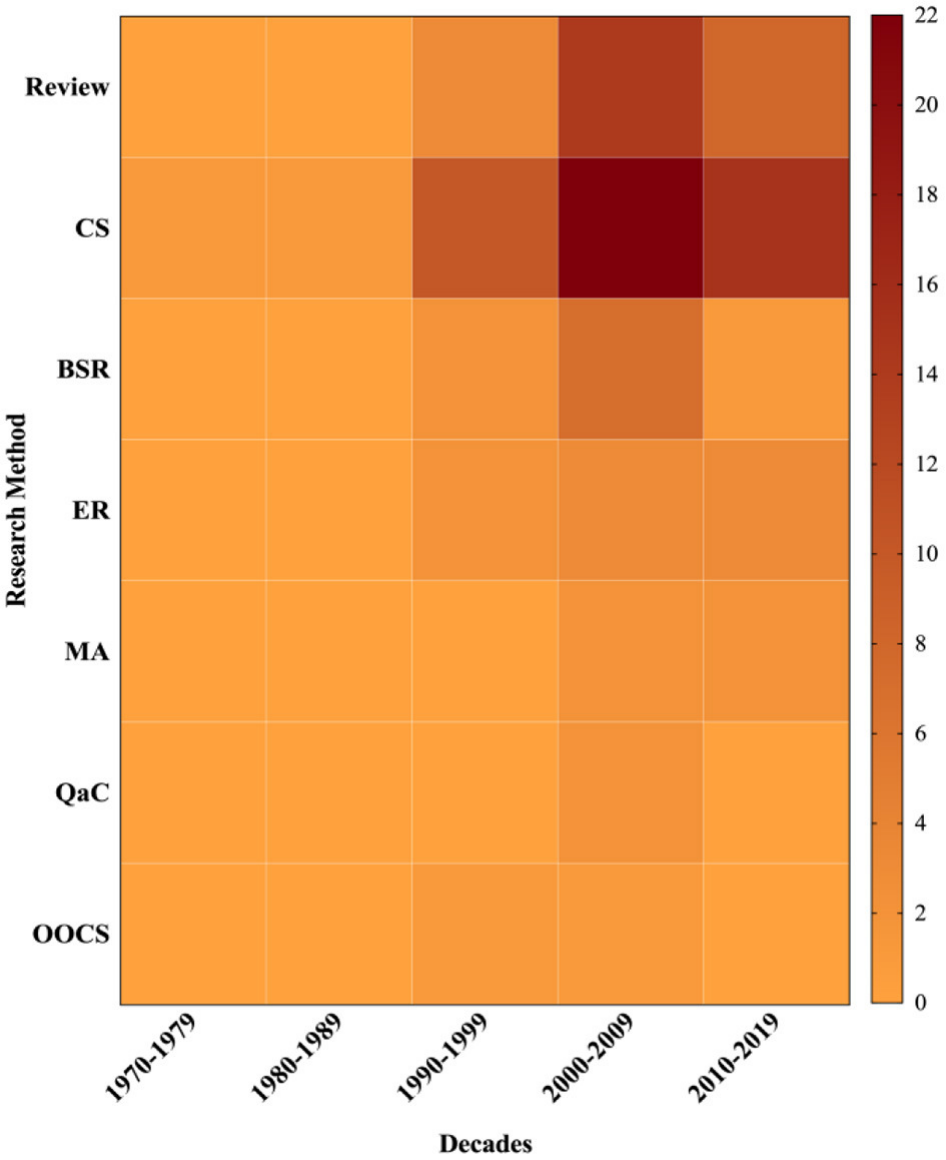
Research methods vs. decades.

**Figure 7:**
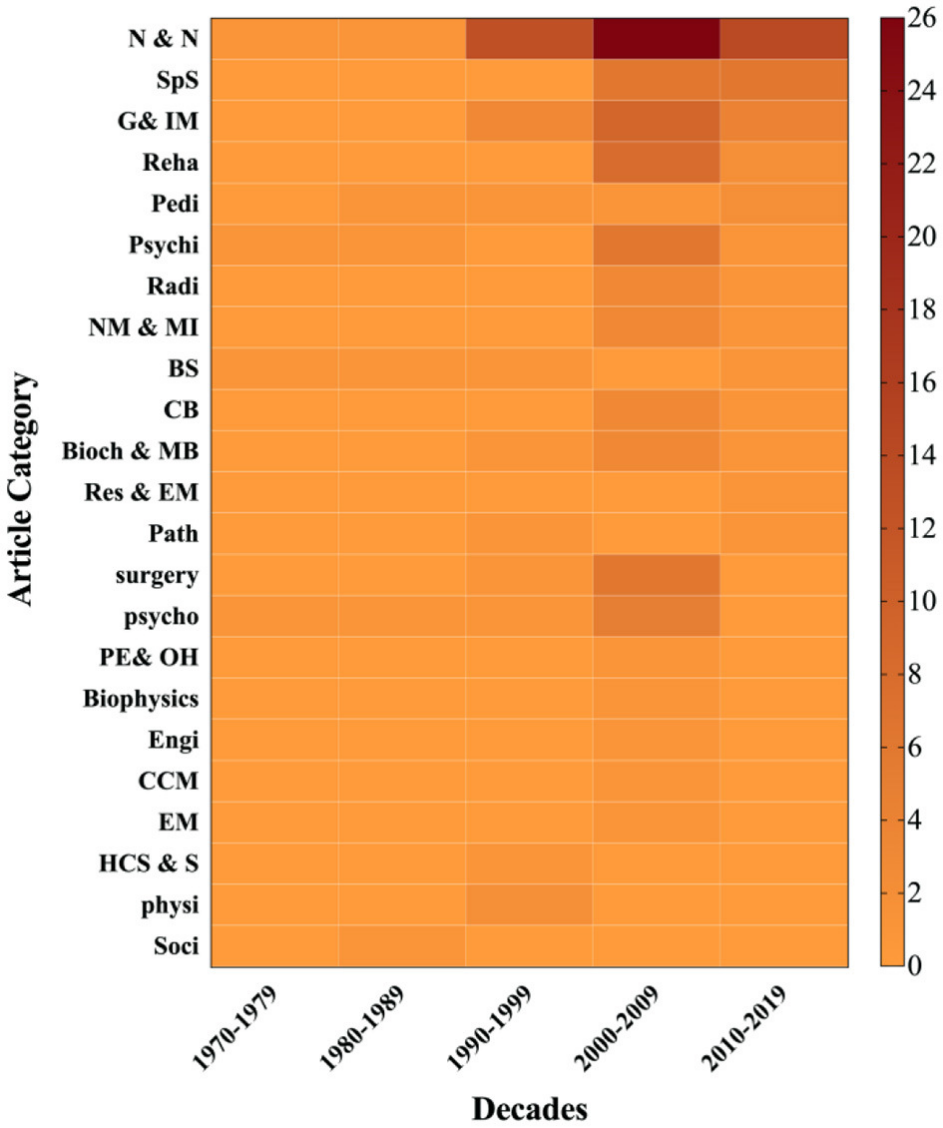
Article categories vs. decades.

The majority of the topics of the top 100 most-cited publications in the primary field study on mTBI were on neurology and neurosciences, followed by those on radiology, psychiatry, pediatrics, rehabilitation, internal and general medicine, and sport sciences, amongst others. A total of 23 categories (the journal categories were not mutually exclusive) can be found and shown in Table 7. The relationship between research methods and publication year, also the article categories and publication year, are shown in Figures 6, 7.

**Table 7:** Article category of the top-100 cited articles.

**Category**	**2010–2019**	**2000–2009**	**1990–1999**	**1980–1989**	**1970–1979**
Neurosciences and Neurology	14	26	13	1	1
Sport Sciences	6	6	0	0	0
General and Internal Medicine	4	9	3	0	0
Rehabilitation	2	8	0	0	0
Pediatrics	2	1	1	1	0
Psychiatry	1	6	0	1	1
Radiology	1	3	0	0	0
Nuclear Medicine and Medical Imaging	1	3	0	0	0
Behavioral Sciences	1	0	1	1	1
Cell Biology	1	3	0	0	0
Biochemistry and Molecular Biology	1	3	1	0	0
Research and Experimental Medicine	1	0	0	0	0
Pathology	1	0	1	0	0
Surgery	0	6	1	0	0
Psychology	0	5	1	1	1
Public, Environmental and Occupational Health	0	1	0	0	0
Biophysics	0	1	0	0	0
Engineering	0	1	0	0	0
Critical Care Medicine	0	1	0	0	0
Emergency Medicine	0	1	0	0	0
Health Care Sciences and Services	0	0	1	0	0
Physiology	0	0	2	0	0
Sociology	0	0	0	1	0

## Discussion

There is no doubt that citation analysis can provide a large amount of information about journals, authors, institutions, and research directions. It can be used to identify landmark papers and influential journals, and help researchers and clinicians discover the basis, current status, and related progress of research. It not only provides a historical review for scientific research in the mTBI field, but also looks forward to its development trends and prospects (11, 12). Insights are provided into the most popular topics regarding the mTBI research field earlier by bibliometric analysis, along with the determination of the characteristics of the specific topics, and the recognition of the major advances in biomedical research. The 100 top-cited publications were cited between 274 and 1,748 times during the period from 1946 to 2020. The changing trends in the research on mTBI over the past 74 years were reflected from the list of the identified publications. Nonetheless, certain meaningful observations and results could be determined, though it being almost impossible to conduct an elaborate analysis of all the publications that were top-cited. The pertinent features of the influential publications in mTBI over the 74 years have been summarized by this study.

The major part of the most-cited publications in the mTBI field was from the United States as compared to that from any other country, which clearly indicated that the publications published from the United States were of high quality and standards over the past five decades. The most top-cited paper focused on the mTBI soldier returned from the Iraq war (13), which provides a great amount of first-hand data to mTBI research understanding. Other top-cited papers concern to a long-term cognitive dysfunction, chronic traumatic encephalopathy, the recovery time following concussion in collegiate football players (14).

The worldwide productivity of published publications on mTBI were concentrated in Australia, Northern Europe, and Northern America, as was evident from the distribution map. JAMA-Journal of The American Medical Association published the most mTBI research worldwide, followed by the New England Journal of Medicine with the highest impact factor. In the United States, the author and research group of the highly cited papers mainly come from comprehensive universities, both the eastern and the west coast of the US. In Australia and Northern Europe, the top mTBI research group publications in the hospital and medicine center.

For a long time, the clinical study predominated the mTBI research, from the 1990's, the review paper attracted more and more attention. Compared to other disease research fields, the proportion of basic science in mTBI was obviously lower (15), indicating that the mTBI research field does have an insufficient accumulation in the injury molecular mechanism study. To some extent, this insufficiency restricted the improvement of early diagnosis and treatment and becomes the bottleneck of mTBI research and development. In terms of mTBI research classification and chronological distribution, the highest cited papers in mTBI published in the early decade of this century, neurosciences & neurology and sport sciences are the main categories, behavioral psychology, surgery, and bioengineering are also involved. In the last decade, the high cited paper has almost decreased by half, which might be related to the absence of the latest breakthrough in the injury molecular mechanism study.

This bibliometric study also has some limitations. Firstly, the mTBI-related publications are being continually cited, the citation metrics might change since we completed the analyses. Secondly, self-reference bias should be should be taken into consideration. Nevertheless, given this research's breadth, richness, and abundant citations, we believe that it can still be invaluable in representing the overall tendency of important publications in the field of mTBI research.

## Conclusions

Improved recognition of the research in the field of traumatic brain injury and a cross-sectional summary of significant research earlier were enabled by the bibliometric analysis of the 100 top-cited publications. Definitely, it would reveal the trends of research requiring development and further investigations besides accelerating the progress of the studies on mTBI.

## Data Availability Statement

The data used to support the findings of this study are available from the corresponding author upon request.

## Author Contributions

XD and MC screened the articles. Data were extracted from each of the articles by JS, XD, and MC. NL, JS, and XD performed the analysis and drafted the manuscript. All authors contributed to the study conception and design, commented on previous versions of the manuscript, read, and approved the final manuscript.

## Conflict of Interest

The authors declare that the research was conducted in the absence of any commercial or financial relationships that could be construed as a potential conflict of interest.
